# Supplementary material for: Intraspecific Diversity and Pathogenicity of Bacillus thuringiensis Isolates from an Emetic Illness
Source: Toxins (Basel). 2023 Jan 18;15(2):89. doi: 10.3390/toxins15020089 (PMC9963800; doi:10.3390/toxins15020089)
Supplement: Supplementary file 1 [file toxins-15-00089-s001.zip › Figure S1.pdf]

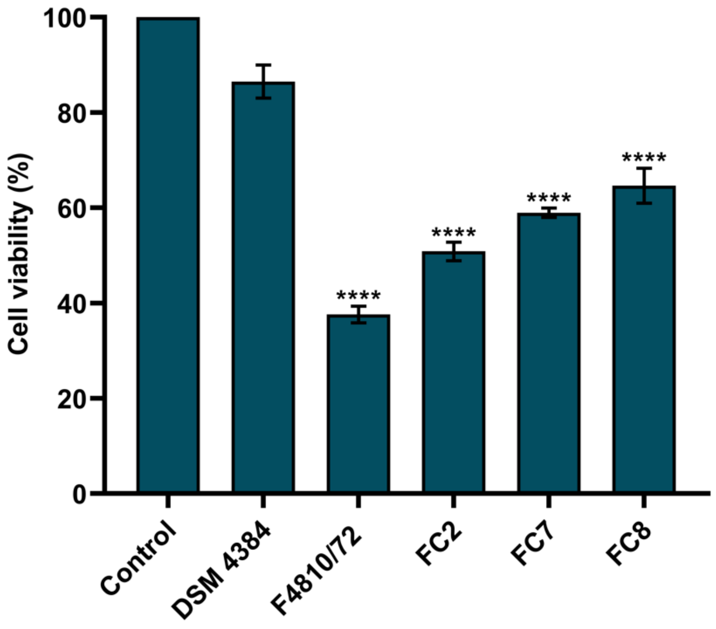

Figure S1. Caco-2 cell viability after treatment with heat-treated culture supernatants of emetic foodborne outbreak isolates. The MTT cytotoxicity test was conducted after 48 hours of exposure to 50% (v/v) (1/2 dilution) of the supernatants of isolates FC2, FC7, and FC8. DSM 4384 was used as a non-emetic strain control, which was non-toxic (cell viability > 80%) to Caco-2 cells. Error bars show ± SD, with n = 3. The asterisk marks indicate a statistically significant difference in cytotoxicity resulting from each emetic strain (reference strain F4810/72 and emetic outbreak FC isolates) as compared to the non-emetic strain *B. cereus* DSM 4384 (one-way ANOVA/Tukey's multiple comparisons test, $p < 0.0001$).
