# Supplementary material for: Intraspecific Diversity and Pathogenicity of Bacillus thuringiensis Isolates from an Emetic Illness
Source: Toxins (Basel). 2023 Jan 18;15(2):89. doi: 10.3390/toxins15020089 (PMC9963800; doi:10.3390/toxins15020089)
Supplement: Supplementary file 1 [file toxins-15-00089-s001.zip › Table S1.pdf]

Table S1. Positive biochemical characteristics of the emetic outbreak *B. thuringiensis* isolates tested using the API 20 and API 50 CH galleries.

**Isolate**	**Positive biochemical characteristics**[a]																			
	Arginine dihydrolase	Citrate (Simmons')	Tryptophan deaminase	Indole	Voges Proskauer	Gelatin liquefaction	Nitrate reduction	Acid from Glycerol	D-Ribose	D-Glucose	D-Fructose	N-acetylglucosa	Amygdalin	Arbutin	Esculin	Salicin	D-Cellobiose	D-Maltose	D-Trehalose	Starch	Glycogen
FC1	5	0	1	1	1	5	5	1	5	5	4	5	0	2	5	4	1	5	5	5	5
FC2	5	2	1	1	1	5	3	1	4	5	5	5	0	3	4	4	1	5	5	5	5
FC6	5	3	1	0	3	5	3	1	4	5	5	5	1	5	5	5	5	5	5	5	5
FC7	5	1	1	0	3	5	5	1	5	5	5	5	1	5	5	5	4	5	5	5	5
FC8	5	2	1	0	2	5	5	1	5	5	5	5	1	5	5	5	4	5	5	5	5
FC9	5	2	1	0	3	5	4	1	4	5	4	5	1	5	5	5	4	5	5	5	5
FC10	5	3	1	0	4	5	5	1	4	5	5	5	1	5	5	5	4	5	5	5	5

[a]Scale recorded show the strength of reaction (5: strongest; 1: weakest; 0: negative); Tests scoring negative for all isolates are not listed.
